# Implementation and evaluation of crowdsourcing in global health education

**DOI:** 10.1186/s41256-022-00279-7

**Published:** 2022-12-15

**Authors:** Huanle Cai, Huiqiong Zheng, Jinghua Li, Chun Hao, Jing Gu, Jing Liao, Yuantao Hao

**Affiliations:** 1 Department of Medical Statistics, School of Public Health, Sun Yat-Sen University, No.74 Zhongshan 2nd Road, Guangzhou, 510080 China; 2 Sun Yat-Sen Global Health Institute, Sun Yat-Sen University, Guangzhou, China; 3 Center for Health Information Research, Sun Yat-Sen University, Guangzhou, China; 4 Peking University Center for Public Health and Epidemic Preparedness and Response, Peking University, Beijing, China

**Keywords:** Global health, Crowdsourcing, Teaching model, Effect evaluation

## Abstract

**Background:**

Current global health course is most set as elective course taught in traditional teacher-taught model with low credit and short term. Innovate teaching models are required. Crowdsourcing characterized by high flexibility and strong application-orientation holds its potential to enhance global health education. We applied crowdsourcing to global health teaching for undergraduates, aiming to develop and evaluate a new teaching model for global health education.

**Methods:**

Crowdsourcing was implemented into traditional course-based teaching via introducing five COVID-19 related global health debates. Undergraduate students majoring in preventative medicine and nursing grouped in teams of 5–8, were asked to resolve these debates in reference to main content of the course and with manner they thought most effective to deliver the messages. Students' experience and teaching effect, were evaluated by questionnaires and teachers’ ratings, respectively. McNemar's test was used to compare the difference in students' experience before and after the course, and regression models were used to explore the influencing factors of the teaching effect.

**Results:**

A total of 172 undergraduates were included, of which 122 (71%) were females. Students' evaluation of the new teaching model improved after the course, but were polarized. Students’ self-reported teaching effect averaged 67.53 ± 16.8 and the teachers’ rating score averaged 90.84 ± 4.9. Students majoring in preventive medicine, participated in student union, spent more time on revision, and had positive feedback on the new teaching model tended to perform better.

**Conclusion:**

We innovatively implemented crowdsourcing into global health teaching, and found this new teaching model was positively received by undergraduate students with improved teaching effects. More studies are needed to optimize the implementation of crowdsourcing alike new methods into global health education, to enrich global health teaching models.

**Supplementary Information:**

The online version contains supplementary material available at 10.1186/s41256-022-00279-7.

## Background

Global health has attracted wide attention worldwide in many fields [1]. Global health concerns not only the health of people but also the training of health workers [2]. China plays a pivotal role in global health [3], but it lacks systematic global health education and professionals [4]. The Chinese Consortium of Universities for Global Health (CCUGH) was established in 2013 [5], which stands for the development of global health education in China [3]. Nevertheless, only few Chinese universities provide systematic global health education [6]. Most of them use the traditional teacher-taught teaching models, and the courses are set as elective courses with low credit and short-term duration [7, 8]. This form of curriculum can hardly trigger students’ learning interest, let alone practice skills learned after the course [9]. Given global health is a multi-disciplinary course with strong knowledge application-orientation, how to facilitate students to get actively engaged and apply knowledge acquired to understand global health related issues is the key teaching need.

To promote students' engagement and improve understanding, prior studies have suggested applying problem-based learning [10], flipped classroom [11] and participatory teaching [12]. More recently, crowdsourcing as a novel form of solution collection [13], characterized by user-centered, high creativity and flexibility [14], has been used in course delivery [15]. Compared with aforementioned teaching models, crowdsourcing has the advantages of high flexibility and strong application-orientation. Centered on the learning tasks set by the teacher, students are on the drivers’ seats to explore the learning source, learning material, and the best manner to demonstrate their learning outcomes. It can not only hone students' ability to apply theoretical knowledge to solve global health related problems, but also increase their interest in self-driven learning. Crowdsourcing has also been widely used in public health researches to improve diagnosis, surveillance, and education [16], with satisfying results [17]. The extent to which crowdsourcing may promote global health education has yet been explored.

Our study applied crowdsourcing to global health education for undergraduates, aiming to develop and evaluate a new teaching model for global health that can improve students’ engagement and understanding.

## Methods

### Study design

The new teaching model based on the main characteristics of crowdsourcing was applied to a ten-week undergraduate course "Introduction to Global Health" of Sun Yat-sen University from May to July, 2021.

**Fig. 1:**
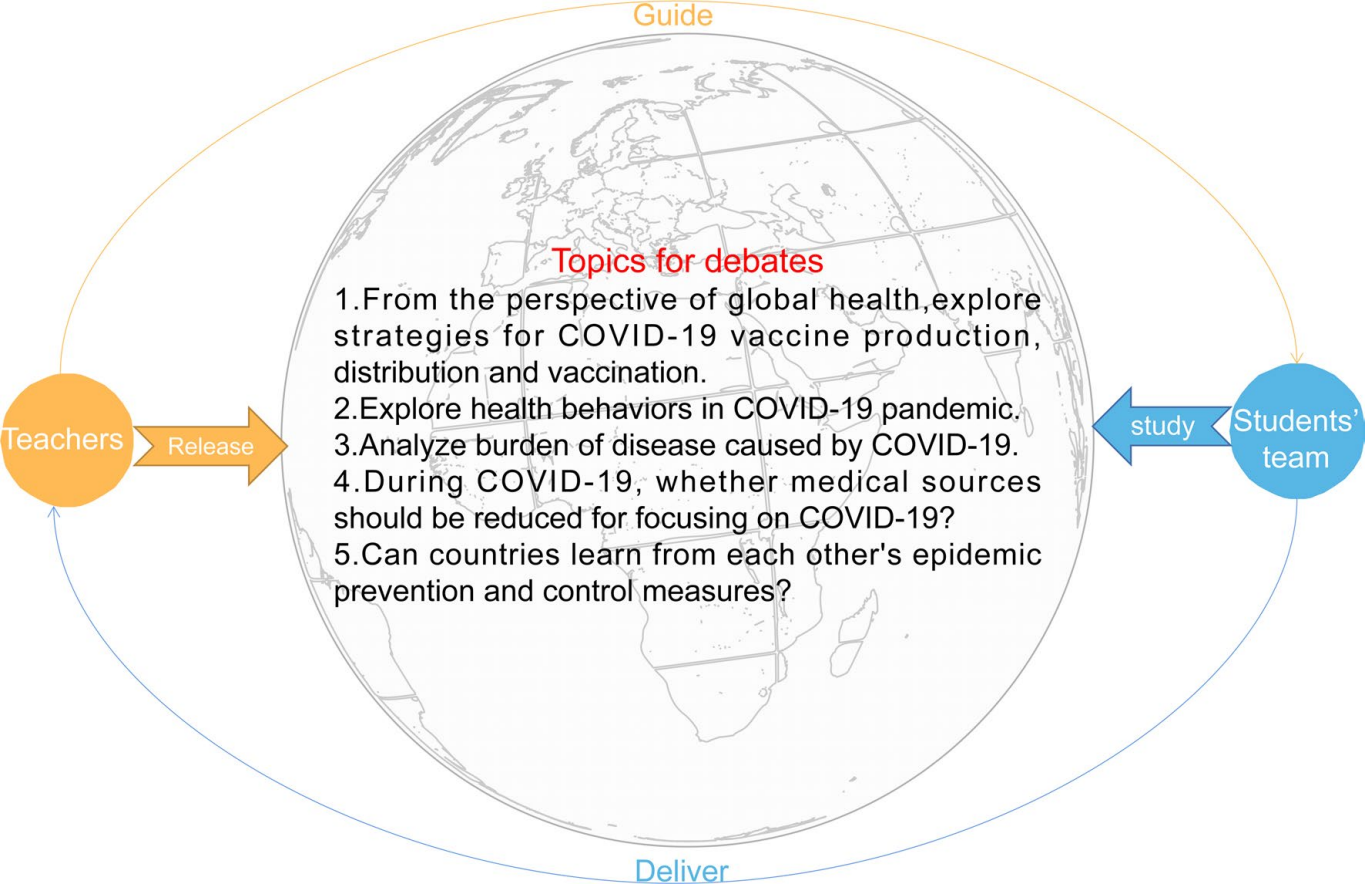
Topics selected for the crowdsourcing-based teaching

**Fig. 2:**
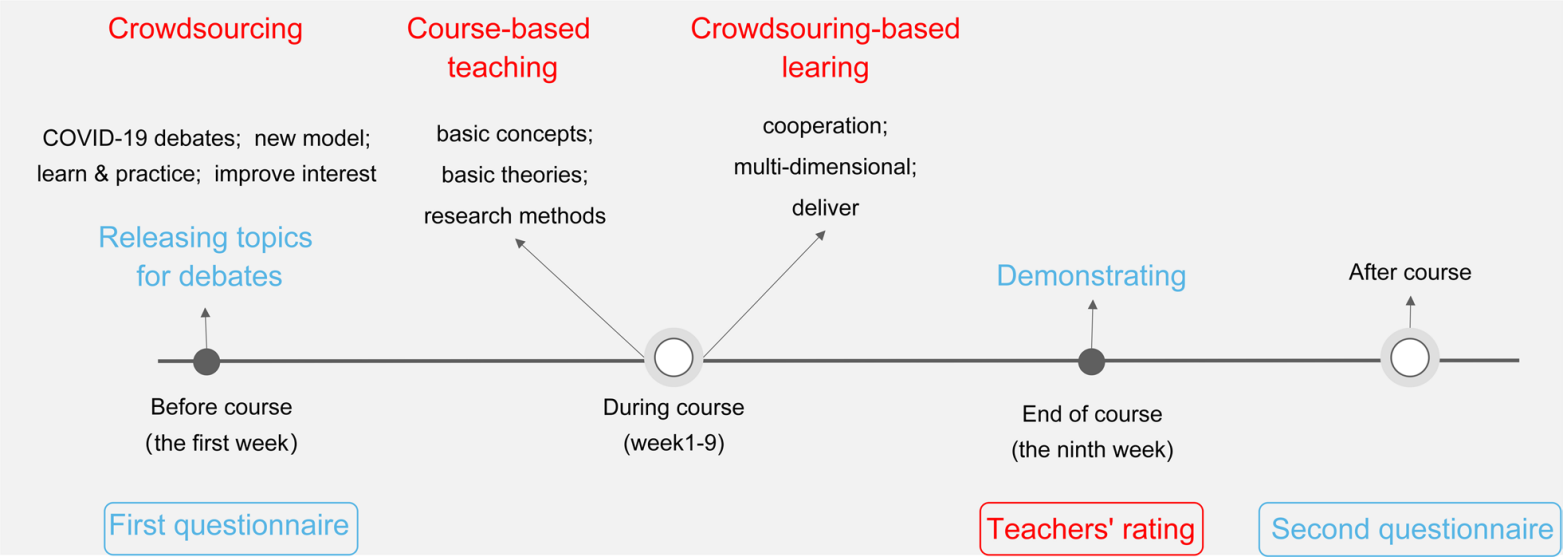
Overall design of the crowdsourcing-based teaching model

The new teaching model consisted of two parts, crowdsourcing debates and traditional course-based teaching. To enhance students' ability to apply what they learned and to increase their interest in learning, we designed a crowdsourcing debates section. In this part, five COVID-19 related global health topics for debates were provided to all students. Students were free to form teams and work on a topic according to their interests. Each team consisted of 5–8 members including a leader. There were 4–6 teams under each topic with a subject teacher supervising them. The teacher organized a discussion among these teams two-weeks before the end of the course. Students can study the topic from multiple dimensions according to their interests and the main content of the course. Then they would choose a manner that they thought most effective to deliver the messages (e.g. video, comedy, debate, etc.) (Additional file 1: Table 1), and some of the demonstrations were interactive (such as interviews). Additionally, we designed a question-and-answer session to increase the participation of other students. Figure 1 shows the topics of crowdsourcing debates. The traditional course-based teaching part was designed to complement students' knowledge of global health. According to the syllabus of “Introduction to Global Health”, basic concepts, theories, and research methods related to global health were taught by teachers every week (Additional file 1: Table 1). The overall design of the new teaching model in this study is shown in Fig. 2.

### Study participants

“Introduction to Global Health” was a compulsory course for undergraduate students majoring in preventive medicine and nursing. Therefore, the participants of our study were 152 students majoring in preventive medicine in grade 2017 and 49 students majoring in nursing in grade 2019, with a total of 201 students. Exclusion criteria: 1) Students who have not completed the course due to suspension or dropping out; 2) Unwilling to cooperate. We obtained the informed consent of all students and the Ethics Committee of the School of Public Health, Sun Yat-sen University has approved this study (#SYSU[2022]NO.085).

### Questionnaire and data collection

We collected data from two aspects. One was students’ self-reported questionnaire before and after the course. The other was the score rated by five teachers based on the same criteria which was for students demonstrating their learning outcomes at the end of the course. We regarded this score as the objective teaching effect. The questionnaire was self-compiled, including basic information, students’ experience, and subjective teaching effect.

Basic information includes gender, major, participation in student union, team leader, team participation, and revision time. This part was investigated only once after the course.

The questionnaire on students’ experience was designed based on the Normalization Process Theory (NPT) [18–20], including an overall dimension and four sub-dimensions. The overall dimension was concerned with students’ overall view of the new teaching model, and sub-dimensions included coherence; cognitive participation; collective action; reflexive monitoring [18]. The specific items of the questionnaire of students’ experience are shown in Additional file 1: Table 2. This part was investigated before and after the course. Items of the overall dimension were calculated with their mean value, and items of four sub-dimensions were discretized into three categories (“agree”, “neutral”, and “disagree”).

The questionnaire on subjective teaching effect was designed on the Kirkpatrick model [21–23]. This part contained a total of 22 items from four dimensions which included reaction, learning, behavior, and results. After calculating the mean value of each dimension, it was summed and converted into a percentage system. This part was investigated only once at the end of the course.

This subjective teaching effect and the objective teaching effect mentioned above together constituted the teaching performance of this study.

### Statistical analysis

We described all the data by using mean ± standard deviation (*Mean* ± *SD*) in quantitative data and the frequency and percentage (*n* (%)) in categorical data. We used Cronbach's *α* coefficient to evaluate the internal consistency of our questionnaire. Its internal consistency was considered to be good if Cronbach's *α* coefficient was over 0.7. McNemar's test (or Fisher's exact probability test) was used to compare the differences of each item of the four sub-dimensions in the parts of the NPT Scale on students’ experience, which were investigated respectively before and after the course. Finally, we regarded subjective and objective teaching effects as outcomes. According to Intraclass Correlation Coefficient (ICC) calculated by linear mixed model, we evaluated whether there was clustering between groups. For lower ICC of subjective teaching effect, multiple linear regression was used to explore its influencing factors. And for higher ICC of objective teaching effect, a linear mixed model was adopted to explore its influencing factors by setting groups as high level and individuals as low level. The Pearson or Spearman correlation coefficients between each influencing factor were 0.15–0.55, and the variance inflation factor (VIF) of each model was less than 5, so we regarded that there was no multicollinearity problem. All statistical analysis was performed in R (V4.1.2) and two-side *P* values < 0.05 was considered statistical significance.

## Results

### Characteristics of participants

There were 201 students in our course and 172 valid questionnaires were collected, with an effective rate of 85.6%. Table 1 shows the characteristics of all participants. We found that 122 (70.9%) were females, 136 (79.1%) majored in preventive medicine, 55 (32.0%) currently participated in student union, 85 (49.4%) formerly participated in student union, and 24 (14.0%) were team leaders. The average level of team participation was 7.90 ± 1.6. The average revision time was 1.42 ± 1.2 h. Cronbach's *α* coefficients of all scales in our questionnaire were about 0.9.

**Table 1:** Characteristics of Participants (*n* = 172)

	*Mean* ± *SD*/*n* (%)
Gender	
Girls	122 (70.9)
Boys	50 (29.1)
Major	
Nursing	36 (20.9)
Preventive medicine	136 (79.1)
Currently participated in student union	
No	117 (68.0)
Yes	55 (32.0)
Formerly participated in student union	
No	87 (50.6)
Yes	85 (49.4)
Team leader	
No	148 (86.0)
Yes	24 (14.0)
Team Participation(10.00 in total)	7.90 ± 1.6
The overall dimension of the NPT Scale before the course(*α* = 0.87) (10.00 in total)	5.79 ± 2.0
The overall dimension of the NPT Scale after the course(*α* = 0.91) (10.00 in total)	6.35 ± 2.2
Revision time (h)	1.42 ± 1.2
subjective teaching effect (*α* = 0.95) (100.0 in total)	67.53 ± 16.8
objective teaching effect (100.0 in total)	90.84 ± 4.9

α: Cronbach's *α* coefficient

### Students’ experience

**Fig. 3:**
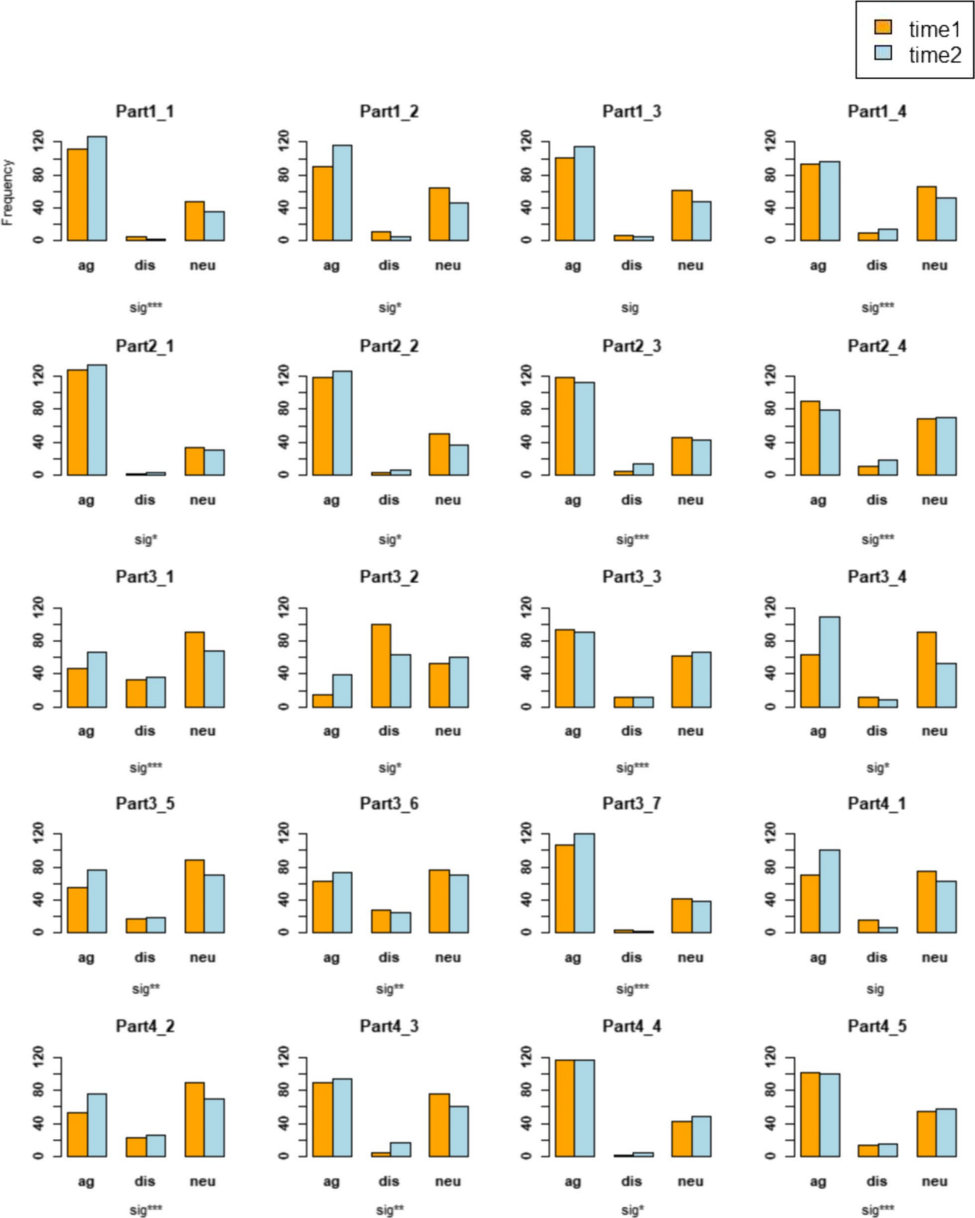
Students’ evaluation of the crowdsourcing-based teaching model before and after the course, based on NPT scale dimension-specifically. Notes： NPT, Normalization Process Theory, which has four dimensions (i.e. coherence, cognitive participation, collective action and reflexive monitoring) noted as Part 1 to Part 4 in the figure. Each dimension contains several questions, noted by the item number. ag: agree, dis: disagree, neu: neutrality. sig*: *P* < 0.05, sig**: *P* < 0.01, sig***: *P* < 0.001

The overall dimension of NPT Scales before and after the course were 5.79 ± 2.0 and 6.35 ± 2.2 respectively, and the McNemar's test showed statistically significant differences (*P* < 0.001). Students’ experience after the course was improved compared with that before the course. Figure 3 shows the investigations before and after the course of each item of the four sub-dimensions. The first sub-dimension was the understanding of the new teaching model. And the number of students who chose “agree” in both cases were more. The second sub-dimension was the participation of the new teaching model. The number of students who chose “agree” in both cases were more, but some students didn’t continue to support the new teaching model after the course. The third sub-dimension was the coordination between the new teaching model and other courses. We found that there was a polarization of students’ ability to adapt to the new teaching model, and the relationship between some students might be disturbed. The fourth sub-dimension was the feedback on the effect of the new teaching model. The number of students who chose “agree” or “neutral” in both cases were more, and students' recognition of the effect of the new teaching model was also polarized.

### Teaching effects and influencing factors

The average score for subjective teaching effect was 67.53 ± 16.8, and the average score for objective teaching effect was 90.84 ± 4.9. And the regression results of influencing factors are shown in Table 2. The regression results of the subjective teaching effect showed that the average score of the teaching effect increased by 2.27 (*P* = 0.004) for each additional 1 h of revision time, and increased by 4.90 (*P* < 0.001) when the average score of the overall dimension in NPT Scale after the course increased by 1. With ICC of 83.5%, the result of linear mixed model showed that the objective teaching effect were 0.78 higher for preventive medicine than nursing (*P* = 0.045), 0.79 higher for students who formerly participated in student union than those who didn’t (*P* = 0.016), and 2.16 higher for team leaders than members (*P* < 0.001). We also found that the average score of objective teaching effect increased by 0.31 (*P* = 0.001) when the average score of team participation increased by 1.

**Table 2:** The regression results of influencing factors for teaching effects

	Subjective teaching effect^I^			Objective teaching effect^II^		
	*β*	95% *CI*	*P*	*β*	95% *CI*	*P*
Gender(boys)	− 0.28	(− 4.02, 3.47)	0.885	− 0.01	(− 0.78,0.77)	0.985
Major(preventive medicine)	− 0.31	(− 4.85, 4.22)	0.892	0.78	(0.04,1.51)	0.045
Currently participated in student union	− 1.65	(− 5.76, 2.46)	0.430	0.17	(− 0.46,0.81)	0.602
Formerly participated in student union	2.40	(− 1.53, 6.33)	0.230	0.79	(0.17,1.40)	0.016
team leader	− 0.49	(− 5.81, 4.83)	0.856	2.16	(1.38,2.94)	< 0.001
Revision time	2.27	(0.74, 3.79)	0.004	0.15	(− 0.09,0.39)	0.224
team participation	1.00	(− 0.20, 2.20)	0.103	0.31	(0.13,0.49)	0.001
Overall dimension of NPT Scale before the course	0.70	(− 0.36, 1.75)	0.195	− 0.01	(− 0.16,0.15)	0.943
Overall dimension of NPT Scale after the course	4.90	(3.91, 5.88)	< 0.001	− 0.02	(− 0.17,0.12)	0.754

Model I: multiple linear regression model; Model II: linear mixed model

## Discussion

We applied crowdsourcing to global health courses, developed and evaluated a new teaching model for global health education. We found that students' feedbacks towards this new teaching model improved after the course. However, there was polarization in students' adaptability to this model. As for teaching effect, students who majored in preventive medicine, formerly participated in student union, were team leaders, spent more time on revision, had higher team participation, and had positive feedback on the new teaching model tended to perform better.

We designed a new global health teaching model based on crowdsourcing, which made full use of its advantages in strong initiative, high flexibility and application-orientation. Previous studies explored different teaching approaches in global health education. For example, the teacher-taught model attached with a World Health Chart could improve the application ability of students in Sweden [24]. In addition to lectures, students were encouraged to communicate with experienced doctors in UK [24]. But these teaching practices were all teacher-centered, and students passively accepted knowledge with poor autonomy. Recently, McNabb et al. provided some strategies based on e-learning tools for global health education under the pandemic of the COVID-19, yet heavily relied on Internet technology [25]. Crowdsourcing, has increasingly been used in teaching field [15]. Geng et al. developed a Crowdsourcing Collaborative Learning Strategy (CCLS) by integrating crowdsourcing and personalized learning in nursing teaching. On the CCLS, students can propose their questions and answer others' questions to learn and obtain customized knowledge from teachers. Compared with the traditional teacher-centered lecture approach, this model had achieved better results [13]. Crowdsourcing can also improve the traditional teaching model by collecting students' questions and targeted teaching [15]. Similarly, Bow et al. developed a platform based on crowdsourcing where students can submit questions and answers and share them with others. Compared with the previous year, students' performance had improved [26]. Generally, there is a lack of authoritative and reliable teaching materials for a new curriculum as global health. Through crowdsourcing, resources can be widely obtained from the students. This method also provides a new idea for curriculum development [27]. Additionally, teachers can rate students more fairly through crowdsourcing [28]. Crowdsourcing can be integrated into education in many forms. However, in these studies, crowdsourcing was mostly used in auxiliary teaching, such as developing platform, collecting learning materials and collecting students' questions. In this model, we fully integrated the course content into crowdsourcing and kept the traditional course-based teaching. The crowdsourcing learning was the main of our course to promote the teaching performance of global health.

Through the implementation of the crowdsourcing teaching model, students' experience improved but polarized. The NPT scale evaluated students' experience in detail through four sub-dimensions. The first (coherence) and fourth (reflexive monitoring) sub-dimensions were the understanding and the feedback on the effect of the new teaching model. The improvements in these domians may result from students' familiarity and recognization of the teaching model, through participation, which also showed in the second sub-dimension (cognitive participation). After the course, however, there was a relative decline in students’ support, in spite of an overall supportive rating. Some research had shown that compared with the traditional teaching model, students were more supportive (collective action) reflecting new teaching model [29], but some students were dissatisfied with the new teaching model [30]. Through the third sub-dimension of the coordination between the new teaching model and other courses and with other students, we found that there was a polarization of students’ capability, and this model might even affect the relationship between classmates. Therefore, it’s considered that the new teaching model was different from the current other courses, and some students cannot adapt to it well, so they didn’t continue to support this model [31]. Studies have shown that students' differences are the main determinants of education effect, which will produce different responses to teaching [32, 33]. Therefore, there was polarization in students' adaptability, leading to conflicts in groups and unpleasant feelings for students.

Furthermore, we found that the more revision time dedicated to the course, the better the subjective teaching effect. It is considered that the more revision time, the richer the knowledge will acquire, and the better the feedback will be in the self-reported/subjective teaching effect. Additionally, students with better experience tend to report higher subjective evaluation of the teaching effect. In the objective teaching effect, we found that the students majoring in prevention medicine, formerly participated in student union, were team leaders, and had high group participation showed better outcomes. There might be two reasons for the better performance of students majoring in preventive medicine. One is that students majoring in prevention medicine were seniors than students majoring in nursing. The comprehensive ability of students in higher grades is better than in lower grades [34, 35]. The other is that prevention medicine major is more relevant to global health [2]. Students who formerly participated in student union performed better, may be due to their abilities of leadership. They were more likely to be the team leaders or play a major role in the teams, and thus were more likely to gain extra awarded points in the scoring criteria. Team collaboration in the new teaching model also played an important role. Prior studies showed that students prefer team collaboration, turned to achieve better outcomes in innovative teaching models [36]. Good team collaboration could allow excellent individual to drive the whole team to study together and to achieve a better teaching effect.

### Strengths and limitations

In this study, crowdsourcing was applied to the global health teaching model design, combining the advantages of crowdsourcing and the characteristics of global health education. We evaluated students’ experience through NPT from the view of implementation science, and examined the teaching effect by the Kirkpatrick model. These methods were widely used in education [37–42], which generate standardized and valid evaluation on the application of the new teaching model to provide new evidence for global health education.

However, there were still some limitations in this study. Firstly, this study is a first attempt of applying crowdsourcing to global health education, the design of the teaching model only regarded crowdsourcing as the guiding ideology and utilizied some crowdsourcing elements. A feasible teaching model that is more consistent with the form of crowdsourcing needs to be further tried. Secondly, due to ethical consideration, we didn’t set a control group. Instead, the teaching effects and influencing factors were explored through regression models and self-comparison of participants before and after the course. Thirdly, the period of this course was just ten weeks, which was too short to evaluate the teaching model for a longer term.

## Conclusions

We applied crowdsourcing to global health courses for undergraduate students, developed and evaluated a new teaching model for global health education. It is found that most students supported this new teaching model and achieved good results. However, the new teaching model is quite different from traditional ones and some students may not have difficulties to adapt to it. To promote the application of crowdsourcing in global health education, it is necessary to streamline the teaching model and highlight the core of global health and crowdsourcing, with better connection of the course content with the teaching model, and improved coordination with other courses. More attention should be paid to the guidance and supplement learning content, encourage students majoring in preventive medicine or formerly participated in student union to play a core role in teams.

## Supplementary Information

**Additional file 1. Supplementary table 1.** The course teaching content and students’ demonstrating form under each issue. **Supplementary table 2.** detail items of all dimensions in NPT Scale

## Data Availability

The datasets generated during and/or analyzed during the current study are available from the corresponding author on reasonable request.

## Abbreviations

CCLS: Crowdsourcing collaborative learning strategy
CCUGH: Chinese Consortium of Universities for Global Health
ICC: Intraclass correlation coefficient
NPT: Normalization process theory
SYSU: Sun Yat-sen University
