# Supplementary material for: Implementation and evaluation of crowdsourcing in global health education
Source: Glob Health Res Policy. 2022 Dec 15;7:50. doi: 10.1186/s41256-022-00279-7 (PMC9753011; doi:10.1186/s41256-022-00279-7)
Supplement: Supplementary file 1 — Additional file 1. Supplementary table 1. The course teaching content and students’ demonstrating form under each issue. Supplementary table 2. detail items of all dimensions in NPT Scale [file 41256_2022_279_MOESM1_ESM.docx]

**Supplementary Material**

Supplementary table 1. The course teaching content and students’ demonstrating form under each issue.

| **Issue** | **Content** | **Example of demonstration**  **form** |
| --- | --- | --- |
| From the perspective of global health,exploring strategies for COVID-19 vaccine production, distribution and vaccination. | Introduction to global health (week1),  Health system responses to global health problems (week4),  Institutions of global health (week8) | Interactive video, sketch comedy |
| Exploring health behaviors in COVID-19 pandemic. | Major health issues in global health (week2),  Major health issues in global health (week9) | Sketch comedy, video |
| Analyzing burden of disease caused by COVID-19. | Research approaches to global health (week6),  Research approaches to global health (week7) | Mock conference, video |
| During COVID-19, whether medical sources should reduce for focusing on COVID-19? | Global health inequalities: Socio-economic determinants of health (week3) | Debates |
| Can countries learn from each other's epidemic prevention and control measures? | The Belt and Road and global health (week5 ) | Debates |

Supplementary table 2. detail items of all dimensions in NPT Scale

| Dimension | Item | Content | Option |
| --- | --- | --- | --- |
| Overall | overall1 | How familiar are you with the new teaching model? | Continuous, each 1 in 0-10. |
|  | Overall2 | Do you think the new teaching model is part of your learning responsibilities? | Continuous, each 1 in 0-10. |
|  | Overall3 | Would you like other courses to follow this model? | Continuous, each 1 in 0-10. |
|  | Overall4 | Do you think the new teaching model is better than the traditional ones? | Continuous, each 1 in 0-10. |
|  | Overall5 | Are you more open to the new teaching model than the traditional ones? | Continuous, each 1 in 0-10. |
| First: the understanding of new teaching model (coherence) | part1_1 | I understand the difference between the new and traditional teaching models. | "Strongly agree, agree, neutral, disagree, strongly disagree" |
|  | part1_2 | All members of this group have reached a consensus on the purpose of the new teaching model. | "Strongly agree, agree, neutral, disagree, strongly disagree" |
|  | part1_3 | I understand the new teaching model for my learning new requirements. | "Strongly agree, agree, neutral, disagree, strongly disagree" |
|  | part1_4 | I agree with the value of implementing the new teaching model for my study. | "Strongly agree, agree, neutral, disagree, strongly disagree" |
| Second: the participation of the new teaching model (cognitive participation) | Part2_1 | There are teachers to motivate us and drive the educational reform project. | "Strongly agree, agree, neutral, disagree, strongly disagree" |
|  | Part2_2 | I think my role in the new teaching model is justified. | "Strongly agree, agree, neutral, disagree, strongly disagree" |
|  | Part2_3 | I am willing to try new learning methods under the new teaching model with group members. | "Strongly agree, agree, neutral, disagree, strongly disagree" |
|  | Part2_4 | I will continue to support the implementation of the new teaching model. | "Strongly agree, agree, neutral, disagree, strongly disagree" |
| Third: the coordination between the new teaching model and other courses (collective action) | Part3_1 | The new teaching model can be easily integrated into my current study. | "Strongly agree, agree, neutral, disagree, strongly disagree" |
|  | Part3_2 | The new teaching model disturbs the existing relationship with classmates | "Strongly agree, agree, neutral, disagree, strongly disagree" |
|  | Part3_3 | I have confidence in the group's ability to accept the new teaching model. | "Strongly agree, agree, neutral, disagree, strongly disagree" |
|  | Part3_4 | The current division for the new teaching model matches our skills. | "Strongly agree, agree, neutral, disagree, strongly disagree" |
|  | Part3_5 | Adequate guidance is provided to enable us to accept the new teaching model. | "Strongly agree, agree, neutral, disagree, strongly disagree" |
|  | Part3_6 | There are sufficient resources to support the implementation of the new teaching model. | "Strongly agree, agree, neutral, disagree, strongly disagree" |
|  | Part3_7 | Teachers fully support the new teaching model. | "Strongly agree, agree, neutral, disagree, strongly disagree" |
| Fourth: the feedback on the effect of the new teaching model (reflexive monitoring) | Part4_1 | I understand the effects of the new teaching model. | "Strongly agree, agree, neutral, disagree, strongly disagree" |
|  | Part4_2 | All members of the group agree that the new teaching model is worthwhile. | "Strongly agree, agree, neutral, disagree, strongly disagree" |
|  | Part4_3 | I attach great importance to the influence of the new teaching model on my study. | "Strongly agree, agree, neutral, disagree, strongly disagree" |
|  | Part4_4 | The group can improve the further implementation of the project based on teacher guidance. | "Strongly agree, agree, neutral, disagree, strongly disagree" |
|  | Part4_5 | I can adjust my learning style according to the requirements of the new teaching model. | "Strongly agree, agree, neutral, disagree, strongly disagree" |

Note:According to the NPT, each dimension corresponds to the overall, coherence, cognitive participation, collective action, reflexive monitoring of the NPT.
